# A novel recurrent *ARL3* variant c.209G > A p.(Gly70Glu) causes variable non-syndromic dominant retinal dystrophy with defective lipidated protein transport in human retinal stem cell models

**DOI:** 10.1093/hmg/ddaf029

**Published:** 2025-03-03

**Authors:** Julio C Corral-Serrano, Veronika Vaclavik, Stijn Van de Sompele, Karolina Kaminska, Katarina Jovanovic, Pascal Escher, Filip Van den Broeck, Francesca Cancellieri, Vasileios Toulis, Bart P Leroy, Julie de Zaeytijd, Zhixuan You, Daniele Ottaviani, Mathieu Quinodoz, Gabriela Bordeanu, Alison J Hardcastle, Frauke Coppieters, Viet H Tran, Michael E Cheetham, Carlo Rivolta, Elfride De Baere

**Affiliations:** UCL Institute of Ophthalmology, EC1V 9EL London, United Kingdom; Unité d'oculogénétique, Jules Gonin Eye Hospital, University of Lausanne, 1004 Lausanne, Switzerland; Center for Medical Genetics, Ghent University Hospital, 9000 Ghent, Belgium; Department of Biomolecular Medicine, Ghent University Hospital, 9000 Ghent, Belgium; Institute of Molecular and Clinical Ophthalmology Basel (IOB), 4031 Basel, Switzerland; Department of Ophthalmology, University of Basel, 4056 Basel, Switzerland; UCL Institute of Ophthalmology, EC1V 9EL London, United Kingdom; Department of Ophthalmology, Inselspital, Bern University Hospital, University of Bern, 3010 Bern, Switzerland; Department of BioMedical Research, University of Bern, 3008 Bern, Switzerland; Department of Ophthalmology, University Hospital Ghent, 9000 Ghent, Belgium; Department of Head and Skin, University Ghent, 9000 Ghent, Belgium; Institute of Molecular and Clinical Ophthalmology Basel (IOB), 4031 Basel, Switzerland; Department of Ophthalmology, University of Basel, 4056 Basel, Switzerland; UCL Institute of Ophthalmology, EC1V 9EL London, United Kingdom; Centro de Investigación Biomédica en Red de Enfermedades Raras (CIBERER), Instituto de Salud Carlos III, Departament de Genètica, Microbiologia i Estadística, Universitat de Barcelona, 08193 Barcelona, Spain; Department of Ophthalmology, University Hospital Ghent, 9000 Ghent, Belgium; Department of Head and Skin, University Ghent, 9000 Ghent, Belgium; Department of Ophthalmology, University Hospital Ghent, 9000 Ghent, Belgium; Department of Head and Skin, University Ghent, 9000 Ghent, Belgium; UCL Institute of Ophthalmology, EC1V 9EL London, United Kingdom; UCL Institute of Ophthalmology, EC1V 9EL London, United Kingdom; Department of Biology, University of Padova, 35121 Padova, Italy; Institute of Molecular and Clinical Ophthalmology Basel (IOB), 4031 Basel, Switzerland; Department of Ophthalmology, University of Basel, 4056 Basel, Switzerland; Department of Genetics and Genome Biology, University of Leicester, LE1 7RH Leicester, United Kingdom; UCL Institute of Ophthalmology, EC1V 9EL London, United Kingdom; UCL Institute of Ophthalmology, EC1V 9EL London, United Kingdom; Center for Medical Genetics, Ghent University Hospital, 9000 Ghent, Belgium; Department of Biomolecular Medicine, Ghent University Hospital, 9000 Ghent, Belgium; Unité d'oculogénétique, Jules Gonin Eye Hospital, University of Lausanne, 1004 Lausanne, Switzerland; Centre for Gene Therapy and Regenerative Medicine, King’s College London, WC2R 2LS London, United Kingdom; UCL Institute of Ophthalmology, EC1V 9EL London, United Kingdom; Institute of Molecular and Clinical Ophthalmology Basel (IOB), 4031 Basel, Switzerland; Department of Ophthalmology, University of Basel, 4056 Basel, Switzerland; Department of Genetics and Genome Biology, University of Leicester, LE1 7RH Leicester, United Kingdom; Center for Medical Genetics, Ghent University Hospital, 9000 Ghent, Belgium

**Keywords:** inherited retinal disease, retinal organoids, ARL3, retinal dystrophy, cilia

## Abstract

Inherited retinal dystrophies (IRDs) are characterized by their high clinical and genetic heterogeneity. Despite significant advances in the identification of genes associated with IRDs, many individuals and families still have not received a definite molecular diagnosis. Here, we performed clinical examinations and conducted genetic testing in five families with IRD. Whole exome sequencing in the five index cases revealed a heterozygous missense variant, c.209G > A, p.(Gly70Glu) in the *ARL3* gene (NM_004311.4). A *de novo* occurrence was demonstrated in one affected individual and autosomal dominant inheritance in nine affected individuals from four families. Their phenotypes displayed variable expressivity, and ranged from rod-cone to cone-rod dystrophy with photophobia. Human induced pluripotent stem cells (hiPSCs) were generated from dermal fibroblasts from the individual with the *de novo ARL3* variant and were differentiated to retinal pigment epithelium cells (RPE) and retinal organoids. Immunofluorescence analyses in these models showed decreased INPP5E localization within the cilia of RPE and connecting cilia of retinal organoids, as well as reduced PDE6⍺ in the organoid outer segments, suggesting that the p.(Gly70Glu) variant causes IRD by defective lipidated protein transport in photoreceptors and/or RPE. This is the first study of ARL3 dysfunction in human retinal cells, highlighting its importance for retinal homeostasis, as well as a variability in the clinical presentation of *ARL3*-associated IRD.

## Introduction

Inherited retinal dystrophies (IRD) are a heterogenous group of retinal diseases that can lead to blindness due to the loss of function of photoreceptor cells. The prevalence in the population is around 1 in 3450 individuals [1], and the pattern of inheritance may be autosomal recessive, autosomal dominant, or X-linked. Over 290 genes have been associated with IRDs (RetNet, September 2024, https://retnet.org). Nevertheless, novel variants and mechanisms affecting the function of these genes and novel candidate genes continue to be identified [2].

The *ARL3* (ADP-ribosylation factor (Arf)-like 3) gene was first associated with autosomal dominant non-syndromic retinitis pigmentosa (adRP, MIM: 618173) in one individual with a *de novo* heterozygous NM_004311.4:c.269A > G, p.(Tyr90Cys) variant [3]. This finding was later confirmed in a second family bearing the same variant [4]. An additional variant in *ARL3*, c.200A > T, p.(Asp67Val) was later identified in a four-generation family with adRP [5]. The *ARL3* c.212A > C, p.(Gln71Pro) variant was identified in a Chinese family with adRP [6]. Nevertheless, other modes of inheritance and potential allelic and phenotypic heterogeneity have also been reported. The variant c.91A > G, p.(Thr31Ala) was found in a father and son with retinal dystrophy; however, the son also had another more common variant in *trans*, c.353G > T, p.(Cys118Phe), and an earlier onset of disease [7]. In addition, a homozygous c.296G > T, p.(Arg99Ile) variant was associated with recessive cone-rod dystrophy [8]. Furthermore, two different amino acid substitutions at residue Arg149 of ARL3 were identified in cases of Joubert syndrome (MIM: 618161), highlighting that different ARL3 amino acid changes can manifest with distinct phenotypes and inheritance patterns [9].

ARL3 is a microtubule-binding protein that is part of the Arf-like family of small GTPases [10]. GTPases contain a G-protein domain that can be activated by switching from guanine diphosphate (GDP)-binding (inactive form) to guanine triphosphate (GTP)-binding (active form). ARL3 localizes to the centrosome, the primary cilium, and the Golgi, and plays a role in cell division [11]. *Arl3* knock-out mice present a severe ciliopathy phenotype with kidney, liver, pancreas, and retinal dysfunction [12]. In the retina, ARL3 localizes to the connecting cilium of photoreceptors [13] and it participates in photoreceptor ciliogenesis and in lipidated protein transport to the outer segments (OS) [14].

Variants in the ciliary gene *ARL13B* are also associated with Joubert Syndrome [15, 16]. ARL13B acts as a guanine exchange factor (GEF) for ARL3 [17, 18], with ARL2BP and CFAP36 acting as co-GEFs to maintain active ARL3-GTP [19, 20]. *ARL2BP* variants have also been associated with autosomal recessive RP (MIM: 615434) [21]. The ARL3 effectors UNC119 and PDEδ, selectively bind myristoylated and prenylated proteins respectively through their lipid moieties. Variants in *PDE6D* (a gene encoding PDEδ) are linked to recessive Joubert syndrome (MIM: 615665) [22], while knockdown of *unc119c* in zebrafish leads to visual impairment and early-onset retinal dystrophy [23]. Heterozygous variants in UNC119 have been linked to autosomal dominant cone-rod dystrophy (CORD24; MIM: 615518) [24, 25]. Binding of ARL3-GTP to UNC119 or PDEδ stimulates their cargo release [26–30]. ARL3 is recycled out of the cilium by its GTPase activating protein (GAP) RP2 [31] that converts ARL3-GTP to ARL3-GDP. RP2 locates to the plasma membrane and the photoreceptor basal body and is associated with X-linked RP with macular involvement [32–35].

Collectively, these studies have revealed the importance of the ARL3 GTPase cycle for cilia function and photoreceptor homeostasis. In this study, we identified a novel variant in *ARL3*, c.209G > A, p.(Gly70Glu) (named here ARL3-G70E), which causes dominant IRD, with a range of clinical presentations in ten individuals from five families. Patients showed rod-cone or cone-rod dysfunction, with photophobia often being an initial presenting symptom. Patient-derived stem cells were investigated for the first time to study how this variant affects cilia morphology and protein trafficking. We report that this ARL3-G70E novel dominant change is associated with altered cilia and outer segment protein traffic in hiPSCs-derived retinal pigment epithelium (RPE) and retinal organoids.

## Results

### Genetic and clinical investigations of five families with IRD

#### Family 1 (Belgium)

**Figure 1:**
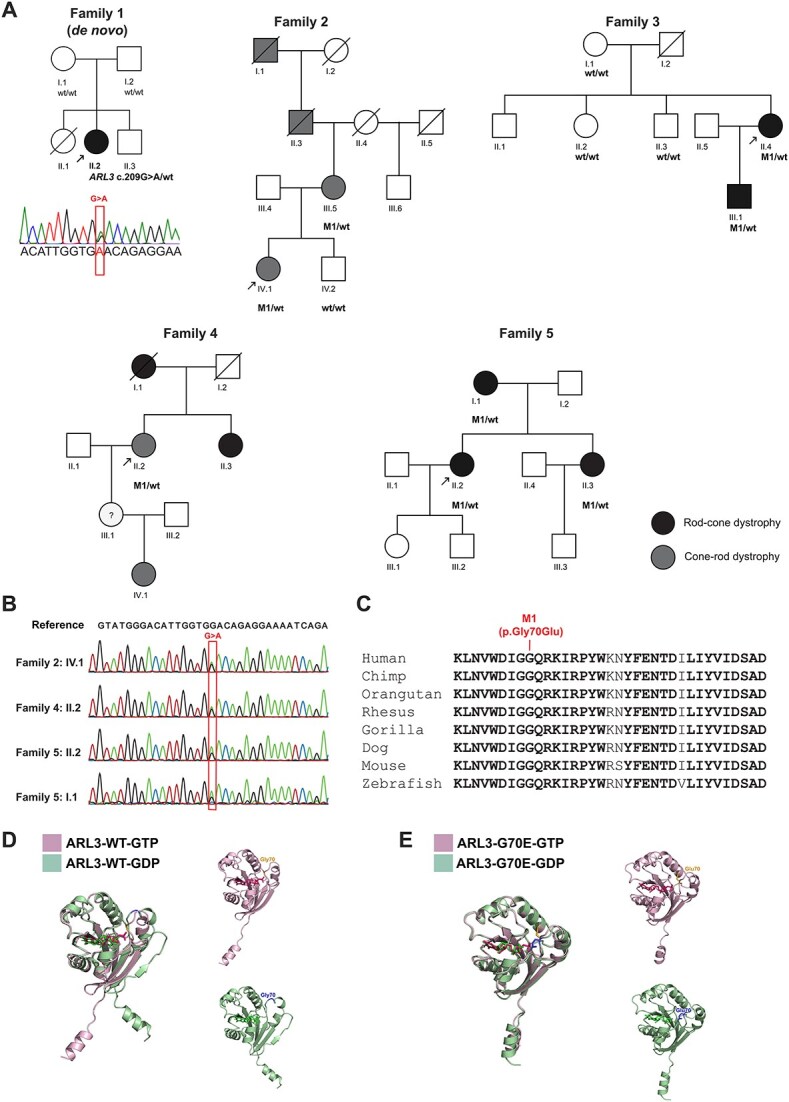
Genetic examination of five families with the *ARL3* c.209G > A variant causing non-syndromic autosomal dominant retinal dystrophy. (A) Pedigrees of five unrelated families showing the affected individuals with the heterozygous change c.209G > A, p.(Gly70Glu) in *ARL3*. (B) Sanger sequencing confirmation of the variant in selected probands and family members. (C) Multispecies protein sequence alignment with conservation of the Gly (G) residue across evolution. Residues that are fully conserved are depicted in bold. M1: *ARL3* c.209G > A, p.(Gly70Glu); WT: Wild-type. (D) AlphaFold prediction of ARL3 wild-type protein (ARL3-WT) structure bound to GTP (pink, top) or GDP (green, bottom), and (E) ARL3-G70E bound to GTP (pink, top) or GDP (green, bottom).

The female patient, individual II.2 (Fig. 1A), was referred to the Ophthalmology Department of Ghent University Hospital at age 21 for genetic testing after a rod-cone dystrophy had been diagnosed. The reason for seeking medical attention was loss of vision, primarily in the dark, and loss of stereopsis. She is the middle child of three, but her elder sibling died before age 2. She is the only one affected in her family (Fig. 1A) and is in otherwise good general health.

#### Genetic investigations of Family 1, individual II.2

The affected individual’s genomic DNA was analyzed by WES-based RetNet panel analysis, which revealed one clinically relevant variant in exon 3 of the *ARL3* gene (NM_004311.3): chr10:g.102699428C > T (GRCh38/hg38), c.209G > A, p.(Gly70Glu). The variant was not present in the gnomAD v.2.1.1 population database, the involved amino acid was well conserved among vertebrates (Fig. 1C), and all the *in-silico* pathogenicity prediction tools scored it as pathogenic/deleterious (MutScore: 0.919, REVEL: 0.98 [36], MetaRNN: 0.99 [37]). Segregation analysis showed that this variant was not present in the parents (Fig. 1A) and was defined as a pathogenic *de novo* change (Table 1).

**Table 1:** Clinical investigations of ten individuals from five families with the *ARL3* variant c.209G > A, p.(Gly70Glu). NA = not available. NR = no detectable response. HM = detection of hand motions.

**Family**	**Individual**	**First symptom**	**BCVA RE/LE (decimal)**	**Age VA**	**Refractive error RE/LE (diopter)**	**Photo-phobia**	**ERG status**	**Fundus appearance**	**OCT**	**VF**
1	II.2	Night-blindness and loss of stereopsis	0.7/0.9	21	−8/−5.75	no	rods: NR, mixed and cones: severly reduced amplitudes	preserved macula with spicular pigmentation in the periphery	largely normal	reduced
2	IV.1	Photo-phobia	0.1/0.32	45	−13/−9	++	rods and mixed: reduced amplitudes, cones and flicker: reduced amplitudes	atrophic perifoveal changes	NA	normal, central scotoma
2	III.5	Reduced visual acuity	HM/0.8	70	NA	no	NA	atrophic changes macula	NA	NA
3	II.4	Night-blindness since teens	0.6/1.25	63	−3.25/−6.25	++/++	rods: NR, mixed: severely reduced, cones: reduced, flicker: NR	atrophic areas nasal	macular pseudo-hole RE	reduced
3	III.1	No symptoms	1.0/1.0	27	−1.75/−2	no	rods, mixed and cones: reduced amplitude	normal appearance	NP	normal
4	II.2	Photo-phobia	0.1/0.1	68	−10.5/−11,5	+++/+++	rods, mixed and cones: reduced amplitudes	peripapillary atrophy, CNV RE,	macular pseudo-hole LE	NA
4	IV.1	Photo-phobia	1.0/0.6	22	−6.75/−7.75	+++/+++	rods and mixed: severely reduced, cones: reduced amplitude	mottled appearance	reduced ONL outside fovea	140°
5	I.1	Night-blindness since 50y	1.25/1.25	54	−1.25/−1	no	NA	bone spicules in periphery	fovea normal	NA
5	II.2	Night-blindness since teens	0.8/0.8	34	−6/−7	yes	NA	bone spicules in periphery	NA	NA
5	II.3	No symptoms	1.25/1.25	32	−4.5/−5	no	rods and mixed: reduced amplitudes, cones: normal?	normal appearance	fovea normal	NA

#### Clinical investigations of Patient 1 (rod-cone dystrophy)

**Figure 2:**
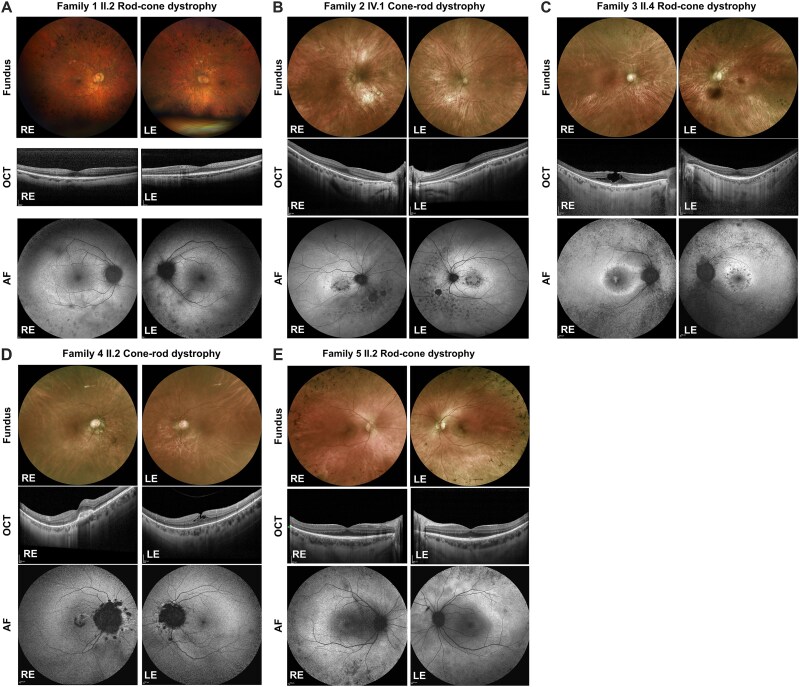
Clinical investigation of all families with the *ARL3* c.209G > A variant causing non-syndromic autosomal dominant retinal dystrophy (A) Fundoscopy of family 1, individual II.2 showing a largely preserved macular area with fine spicular intraretinal pigmentation in the periphery and normal vascular calibers. OCT of the macula showing well-preserved retinal architecture and thickness. Blue-light autofluorescence imaging showing largely preserved macular autofluorescence surrounded by granular hyperautofluorescence. (B) Family 2, individual IV.1. Fundus color imaging shows areas of atrophy, nasal to the optic disc, with an absence of pigmentary changes. OCT shows reduced ONL outside the fovea. AF images show a hypoautofluorescent ring around the fovea and hypoautofluorescent areas nasal to the optic nerve. (C) Family 3 individual II.4. AF images show a hyperautofluorescent ring in the right eye, with a lamellar pseudohole on OCT. the left eye has areas of hypoautofluorescence around the fovea on AF imaging and reduced ONL outside the fovea. (D) Family 4, individual II.2. Fundus color imaging shows areas of atrophy with an absence of bone spicules. OCT of the right eye shows a scar from previous choroidal neovascular membrane (CNV) and left eye a lamellar pseudohole. AF images show hypoautofluorescent areas nasal to the disc, as well as hypoautofluorescence at the fovea. (E) Family 5, individual II.3. Fundus imaging reveals sparse bone spicules. OCT shows reduced ONL outside the fovea. A hyperautofluorescent ring can be seen on AF imaging. AF: Autofluorescence; OCT: Optical coherence tomography; ONL: Outer nuclear layer; RE: Right eye. LE: Left eye.

At the time of examination, she had distant vision of 0.7 and 0.9 (decimal, Snellen notation) with a correction of −8.00 D -0.75 D at 60° and − 5.75 D -0.75 D at 120° for the right and left eye, respectively. Color vision testing detected a very mild red-green color deficiency on Ishihara and HRR pseudo-isochromatic plates. The Farnsworth D-15 color test was normal. Goldmann kinetic visual fields showed constricted peripheral, pericentral and central sensitivities without scotomata. Full-field ERG detected an absence of scotopic responses and severely reduced photopic responses (Table 1). Fundoscopy revealed a largely preserved macular area with fine spicular intraretinal pigmentation in the periphery and normal vascular calibers (Fig. 2A). Blue-light autofluorescence imaging showed largely preserved macular autofluorescence surrounded by granular hyperautofluorescence. Background autofluorescence outside this area was interrupted by spotty hypoautofluorescence (Fig. 2A). On near-infrared-light autofluorescence imaging a macula-centered rhomboid area of preserved autofluorescence signal with a parafoveal ring of mildly reduced signal was visible (Supplementary Fig. S1A). Outside of this rhomboid, the autofluorescence signal was severely reduced. OCT of the macula revealed well preserved retinal architecture and thickness (Fig. 2A). Over the 10-year follow-up, visual function remained remarkably stable, but a slow anatomical deterioration with progressive loss of photoreceptors encroaching the macula area could be observed.

#### Genetic investigations of Families 2–5 (Switzerland)

WES analyses for the probands described below yielded negative results for known pathogenic variants in IRD genes. Therefore, the analysis was expanded to novel variants in such genes, which revealed the same heterozygous missense variant NM_004311.4: c.209G > A, p.(Gly70Glu) in the *ARL3* gene (Fig. 1A), as described before for individual II.2 (Family 1). The variant co-segregated with the disease in families 2–5, where samples from subsequent individuals were available, and was compatible with a dominant transmission of the disease.

#### Clinical investigations of Family 2 (cone-rod dystrophy)

The proband (Fig. 1A, Family 2 individual IV.1) was referred to the Lausanne Ophthalmology Clinic following a severe progression of her myopia in her adult life: she had a correction of −7.5 D in her right eye and − 3.5 D in the left when she was 28 years old. Eleven years later, at the age of 39, she had −11 D in her right eye and − 7.5 D in the left. Her best corrected visual acuity (BCVA) at the last visit (45 years) was 0.1 in the right and 0.3 in the left eye. The affected individual complained of photophobia and poor reading vision. Fundus examination showed areas of perifoveal atrophy as well as areas of atrophy nasal to the optic nerve (Fig. 2B), AF imaging showed a ring of hypoautofluorescence around the fovea (Fig. 2B). Her 70-year-old mother had vision of hand movement in her right eye, and 0.8 in the left. Fundus examination showed extensive atrophy at the macula, and nasal to the optic nerve in the right eye and perifoveal atrophy, with foveal sparing in the left (Supplementary Fig. S1B). Both daughter and mother had the *ARL3* variant c.209G > A, p.(Gly70Glu) heterozygously, while the asymptomatic brother was negative for the variant (Fig. 1A). Following full-field ERG testing the diagnosis of cone-rod dystrophy was confirmed (Table 1).

#### Clinical investigations of Family 3 (rod-cone dystrophy)

The proband (Fig. 1A, Family 3 individual II.4), now 63 years old, noticed night blindness since she was a teenager. Later in life, she had reduced visual fields, while her BCVA remained at 1.25 in the left eye, and 0.6 in the right, due to lamellar macular hole. She was myopic at −3 D in the right and − 6.5 D in the left eye. The fundus and autofluorescence findings showed perifoveal areas with atrophy as well as beyond arcades in the left, and hyperautofluorescent rings in both eyes (Fig. 2C). Full-field ERGs revealed undetectable rod-specific ERGs and severely reduced cone ERGs, consistent with rod-cone dystrophy. When the patient’s asymptomatic 27-year-old son (Fig. 1A, Family 3, individual III.1) was assessed, his BCVA was 1.25 in both eyes, with minor myopic correction (−1 D in both eyes). Full-field ERGs were severely reduced for scotopic and photopic responses. The OCTs showed reduced outer nuclear layer (ONL) thickness outside the fovea in both eyes (Supplementary Fig. S1C). Both affected individuals carried the heterozygous variant c.209G > A, p.(Gly70Glu) in *ARL3*.

#### Clinical investigations of Family 4 (cone-rod dystrophy)

The proband (Fig. 1A, Family 4 individual II.2) of this family always suffered from severe photophobia. She was 49 years old when she first went to her local ophthalmologist, complaining mainly of reduced vision, but no night blindness. Her visual acuity at that time was 0.5 with −8.25 D in the right and 0.4 with −7.75 D in the left eye. Full-field ERG showed mild rod and cone dysfunction, while the Goldmann visual field was severely constricted. Over the following years, her visual acuity continued to decline, despite a bilateral cataract operation she underwent when she was 62 years old. Following the surgery, her photophobia worsened, and she could only leave her house wearing dark sunglasses, extending to the side, and a hat. She also developed a choroidal neovascular membrane in her right eye, which was stabilized with 2 intravitreal injections of anti-VEGF. In her left eye, an early stage of a macular lamellar pseudo hole was noticed. The last recorded visual acuity was 0.1 in both eyes, with almost normal appearance of her fundus. Autofluorescence imaging was difficult to perform, due to her severe photophobia, but some peripapillary atrophy and a large hyperautofluorescent area within arcades (Fig. 2D) could be identified.

The proband’s mother and sister were previously diagnosed with retinitis pigmentosa (RP), but we were not able to perform an ophthalmological assessment on them. Only the proband’s granddaughter (Fig. 1A, Family 4, IV.1) could be ophthalmologically examined as she presented at the clinic with reports of photophobia, and later also of night vision problems. Her BCVA was 1.0 with −6 D in both eyes. The ERGs were subnormal in scotopic and photopic conditions, while Goldmann visual field was within normal limits for isopter V4e, isopter I4e was reduced to 70° bilaterally. The autofluorescence imaging showed a band of hyperautofluorescence beyond the arcades (Supplementary Fig. S1D). Both affected individuals, II.1 and IV.1, harbored the variant c.209G > A, p.(Gly70Glu) in *ARL3* in heterozygous state. As for the proband’s daughter (individual III.1), no medical conclusions could be reached, as she refused to go to the hospital for any assessments or genetic testing.

#### Clinical investigations of Family 5

The proband (Fig. 1A, Family 5, individual II.2) was referred to the Lausanne Ophthalmology Clinic after her private ophthalmologist noticed bone spicules at her fundus (Fig. 2E). The patient also had rapidly progressive myopia since she was 4 years old. When first examined in Lausanne at the age of 25, her BCVA was 1.0 with −5.75 D in the right eye and − 6.75 D in the left. The first ERG testing showed diffuse scotopic and photopic dysfunction, in keeping with rod-cone dystrophy. In her late twenties, she developed severe photophobia, which made it very difficult for her to wear contact lenses. She inquired about refractive surgery, but her request was declined.

The patient’s mother, (Fig. 1A, Family 5, I.1;56 years old), was referred by her private ophthalmologist who had noticed some bone spicules. The patient is mildly symptomatic and is still driving her car. Her autofluorescence image showed a broad ring of autofluorescence, beyond arcades (Supplementary Fig. S1E). Proband’s sister (Supplementary Fig. S1F, Family 5, II.3; 34 years old), who was not complaining of night blindness or photophobia also underwent an ophthalmological assessment. She had obtained a LASIK surgery for myopia (−5 D in both eyes) when she was 22 years old. Following examination and autofluorescence imaging, which revealed a broad ring of hyperautofluorescence (Supplementary Fig. S1F), she underwent ERG analysis. The full-field ERGs were subnormal in photopic conditions (delayed implicit time for 30 Hz Flicker) but normal in scotopic conditions. All 3 affected individuals (the mother and the 2 daughters) carried the c.209G > A, p.(Gly70Glu) variant in *ARL3* in heterozygosity. We are currently following the 2 children of each sister, but, to date, no genetic testing has been performed.

### ARL3-G70E does not affect ciliation or cilium length in fibroblast cells, hiPSCs-RPE, and hiPSCs-RO models

**Figure 3:**
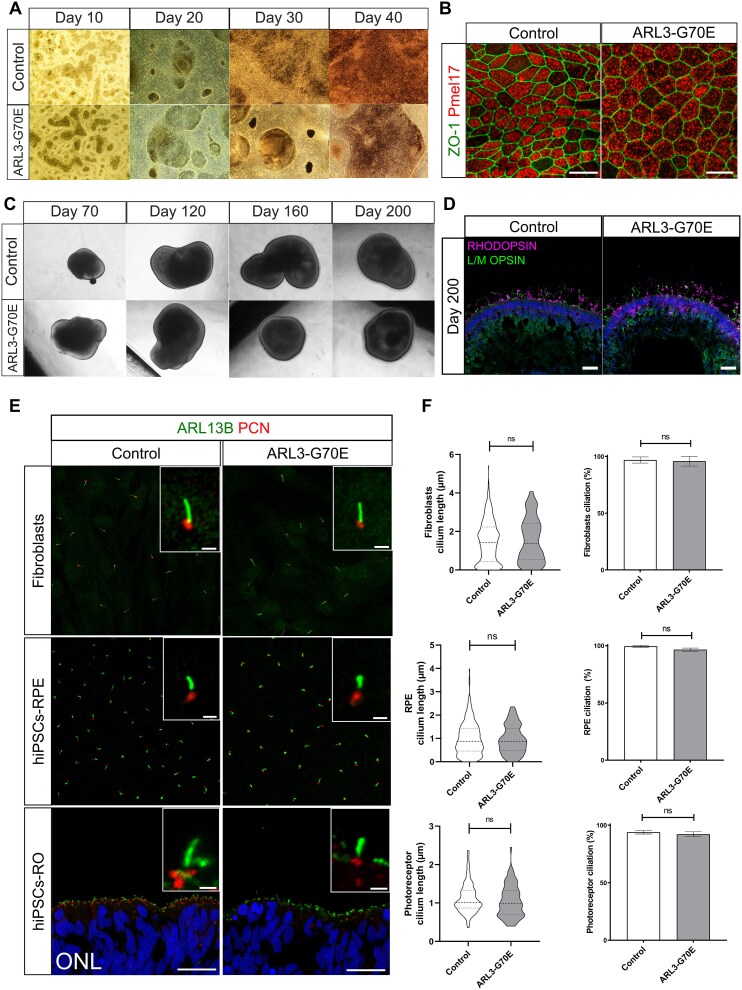
The ARL3-G70E variant does not have a major effect on ciliation or cilium length. (A and B) hiPSCs-RPE differentiation. (A) Bright-field images of control and ARL3-G70E hiPSCs-derived RPE cells (hiPSCs-RPE) during the differentiation process at days 10, 20, 30 and 40. (B) Immunofluorescence analysis of hiPSCs-RPE cells stained with the cell junction marker ZO-1 (green) and the melanosome marker PMEL17 (red). Scale bars: 20 μm. (C and D) hiPSCs differentiation to retinal organoids (hiPSCs-ROs). (C) Bright-field images of control and ARL3-G70E hiPSCs-ROs during the differentiation process at days 70, 120, 160, and 200. (D) Immunofluorescence analysis of ROs at day 200 stained with the rod photoreceptor marker RHODOPSIN (magenta), and the cone photoreceptor marker L/M OPSIN (green). DAPI marks the nuclei (blue). Scale bars: 20 μm. (E) Cilia staining in fibroblasts, hiPSC-RPE, and photoreceptors in hiPSC-ROs, as indicated, with the markers ARL13B (green) and PCN (red). DAPI (blue) marks the photoreceptor nuclei from ROs. Scale bars are 1 μm for insets, and 20 μm for the photoreceptor ONL images. (F) Cilia length was analyzed using CiliaQ as a measure of the ARL13B length and PCN as ciliary base. For fibroblasts, *n* = 373 control cilia and *n* = 148 ARL3-G70E cilia; for RPE, *n* = 285 control cilia and *n* = 112 ARL3-G70E cilia; for ROs, *n* = 93 control cilia and *n* = 77 ARL3-G70E cilia. *P*-value: Ns, not significant, as assessed by Mann–Whitney test.

To study the effect of the ARL3-G70E variant on primary cilia structure and function, fibroblasts were obtained from the individual with the *de novo* variant in *ARL3*, c.209G > A, p.(Gly70Glu) (Fig. 1A, Family 1, individual II.2). Given that the ARL3-G70E variant causes non-syndromic IRD, we next aimed to study the effect of this variant in retinal cells. Therefore, the ARL3-G70E patient fibroblasts were reprogrammed to human induced pluripotent stem cells (hiPSCs) and subsequently differentiated into either retinal pigment epithelium (hiPSC-RPE) cells or retinal organoids (hiPSC-ROs). Both hiPSC-RPE cells and hiPSC-ROs from the ARL3-G70E line differentiated similarly to controls without any obvious morphological abnormalities (Fig. 3A–D).

First, we examined whether there were differences in cilium length in fibroblasts under serum starvation conditions. No significant differences in cilium length or incidence were observed between the control and ARL3-G70E cells (Fig. 3E and F). Similarly, no significant differences were observed for cilia incidence or length in ARL3-G70E hiPSC-RPE cells or photoreceptors in the hiPSC-ROs (Fig. 3E and F).

### ARL3-G70E hiPSCs-RPE and hiPSC-ROs show a reduction of ciliary INPP5E and PDE6⍺

**Figure 4:**
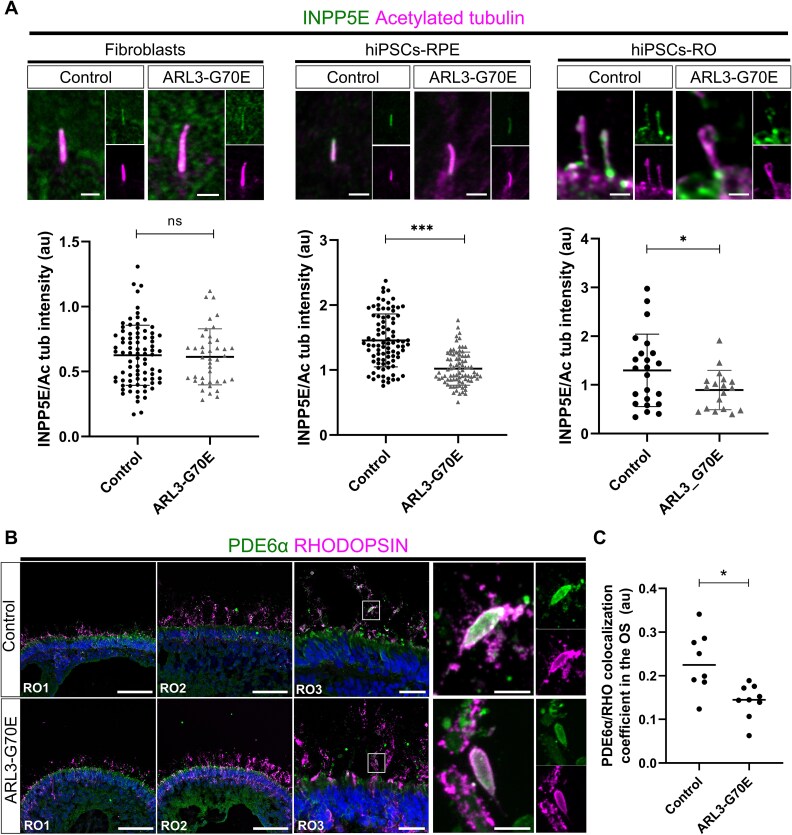
ARL3-G70E hiPSCs-derived RPE (hiPSCs-RPE) and retinal organoids (hiPSCs-ROs) show a reduction of ciliary INPP5E and PDE6⍺. (A) Upper panels: Cilia were stained with INPP5E (green) and acetylated tubulin (magenta) in fibroblasts, hiPSCs-RPE, and photoreceptors of hiPSCs-ROs. hiPSCs-ROs were differentiated for 120 days to examine photoreceptor cilia. Scale bars: 2 μm (fibroblasts), 1 μm (hiPSCs-RPE and hiPSCs-RO). Lower panels: The INPP5E/acetylated tubulin fluorescence intensity ratio was measured. For fibroblasts, *n* = 86 control cilia and *n* = 41 ARL3-G70E cilia; for RPE, *n* = 88 control cilia and *n* = 82 ARL-G70E cilia, for ROs, *n* = 23 control cilia and *n* = 18 ARL3-G70E cilia. Ns, not significant. ^*^P-value < 0.05, ^*^^*^^*^P-value < 0.001 as assessed by Mann–Whitney test. (B) hiPSCs-ROs were differentiated for 200 days and stained with PDE6⍺ (green), rhodopsin (magenta) and DAPI (blue). Scale bars: 100 μm for RO1 and RO2 images; 25 μm for RO3 image, and 5 μm for the RO3 insets. (C) The PDE6α to rhodopsin (RHO) colocalization coefficient in the OS region was analysed using JACoP on ImageJ. For controls, 8 images from *n* = 4 ROs were measured. For ARL3-G70E, 9 images from *n* = 5 ROs were measured. ^*^P-value < 0.05 as assessed by Mann–Whitney test. RO: Retinal organoid. OS: Outer segments.

Previous studies have reported that the level of the prenylated Inositol Polyphosphate-5-Phosphatase E (INPP5E) is reduced in the cilia of fibroblasts from patients carrying *ARL3* variants and presenting with Joubert syndrome [9, 38], suggesting that ARL3 regulates the ciliary membrane localization of INPP5E [9, 22, 39, 40]. To investigate if cilia traffic was disrupted as a consequence of ARL3-G70E, the localization of INPP5E was analyzed in patient-derived fibroblasts, hiPSC-RPE, and hiPSC-ROs. In ARL3-G70E fibroblasts, INPP5E intensity was not different from that of controls (Fig. 4A). Importantly, however, the intensity of INPP5E immunoreactivity was significantly reduced in the cilium of hiPSCs-RPE and the connecting cilium of hiPSCs-ROs (Fig. 4A). The localization of another ARL3 prenylated cargo, PDE6α, was also investigated. There was a general reduction in the immunoreactivity for PDE6α in the outer segments of the ARL3-G70E patient organoids compared to controls (Fig. 4B). This was confirmed by measuring the co-localization of PDE6α with rhodopsin in the organoid outer segments (Fig. 4C), suggesting that ARL3-G70E compromises lipidated protein traffic to the connecting cilium and outer segment.

### ARL3-G70E is stable and not constitutively active

To examine the effect of the G70E variant on ARL3 protein stability and on the interaction with its GAP RP2, co-immunoprecipitation experiments were performed. HEK293T cells were co-transfected with RP2-GFP, and either the ARL3-WT-VSV (wild-type, control), the constitutively active Arl3-GTP conformational mimic ARL3-Q71L-VSV or with ARL3-G70E-VSV plasmids. Western blot images showed that the levels of ARL3-G70E-VSV were comparable to ARL3-WT-VSV, suggesting that the protein is stable and not misfolded (Supplemental Fig. S3). Moreover, the constitutively active form, ARL3-Q71L-VSV, displayed stronger binding to RP2, as previously reported [41]. In contrast, ARL3-G70E-VSV showed no differences in binding to RP2, suggesting that ARL3-G70E is not constitutively active *per se*, even though AlphaFold 3 modelling suggests that ARL3-G70E leads to the GDP bound state to resemble the structure of WT ARL3-GTP (Fig. 1D and E).

## Discussion

### ARL3-G70E leads to non-syndromic dominant retinal dystrophy with complete penetrance and variable expressivity

ARL3 is an important GTPase of the primary cilium [10, 11, 13, 14, 42]. Recently, several studies have described variants in *ARL3* associated with retinal dystrophy and other ciliopathies [3–6, 8, 9]. In this study, we report a novel heterozygous missense variant in ARL3, p.(Gly70Glu) or G70E, which causes autosomal dominant IRD in five different families. Collectively, the evidence supports this variant as pathogenic (Supplementary Table S1). Interestingly, the clinical presentation across these families was highly variable, differing in terms of age of onset, disease progression, and subtype of IRD. Affected individuals from three families (Families 1, 3, and 5) exhibited rod-cone dystrophy with classic RP symptoms, whereas those from two other families (Families 2 and 4) had cone-rod dystrophy with photophobia. The underlying reasons for this clinical heterogeneity among individuals carrying the same *ARL3* variant remain unclear. Intrafamilial variability was also observed in Family 4, where some members presented with cone-rod dystrophy and photophobia, while others had reported symptoms more typical of RP. This suggests that genetic and environmental modifiers could influence both the presentation and progression of the disease. The variant perfectly segregated with the disease, suggesting complete penetrance. Interestingly, all the affected individuals appear to be myopic. Myopia is also frequently associated with pathogenic variants in the ARL3 GAP RP2, which cause X-linked IRD. *RP2*-associated retinopathy is a severe early-onset disease with the majority of cases showing rod-cone dystrophy, but early cone-system disease and macular involvement can vary greatly [35]. The phenotypic variability associated with the *ARL3* c.209G > A, p.(Gly70Glu) variant shows that caution might be required in diagnosing individuals with *ARL3*-associated retinopathy.

### ARL3-G70E leads to a defect in INPP5E and PDE6α ciliary trafficking in hiPSCs-RPE and hiPSCs-ROs models

In addition to genetic and clinical assessments, we investigated the effects of ARL3-G70E in the cilia of patient-derived hiPSCs-RPE and of photoreceptors from hiPSCs-ROs. Interestingly, we identified a reduction in INPP5E cilia localization and PDE6α outer segment trafficking in these models. Conditional knock-out of *Arl3* in mouse photoreceptors results in a strong phenotype with no outer segment formation and rapid photoreceptor degeneration [14], suggesting that ARL3 is essential for cilia formation, photoreceptor cell differentiation, and their maintenance. Different variants in *ARL3* can have different effects on ARL3 function. For example, the ARL3-R149C/H variants are unable to interact with ARL13B, thus remaining in the GDP inactive form. This loss of the ability to activate ARL3 disrupts ciliary protein composition, and is thought to be the molecular mechanism behind *ARL3*-related Joubert syndrome [8, 9]. By contrast, the ARL3-Y90C variant is linked to non-syndromic autosomal dominant RP [3, 4] and was shown to act as a fast-cycling GTPase [43]. Conversely, the ARL3-R99I variant affects ARL3 stability, and is associated with autosomal recessive cone-rod dystrophy, presumably by acting as a hypomorphic allele [8]. The amino acid substitution ARL3-Q71L, designed based on known dominant negative alleles of other small GTPases to act as a conformational mimic of ARL3-GTP, is constitutively active, unable to hydrolyze GTP. Similarly, ARL3-D67V has also been reported to be constitutively active [43]. The presence of ARL3-Q71L in mouse photoreceptors leads to a reduction of PDE6α, PDE6β, and GRK1, caused by sequestration of PDE6δ by ARL3-Q71L [44].

### ARL3-G70E may act as a fast-cycling GTPase

Therefore, it appears that dominant alleles in ARL3 are associated with an altered GTPase cycle that favors ARL3-GTP, similar to the loss of its GAP, RP2. On the other hand, hypomorphic alleles lead to recessive disease, while changes that favor ARL3-GDP lead to syndromic presentation, similar to the loss of ARL13B, as reviewed in [45]. Our modelling with AlphaFold 3 shows that ARL3-G70E bound to GDP resembles the GTP bound structure of ARL3, suggesting the variant favours a GTP-like state (Fig. 1D,E).

It is possible that ARL3-G70E acts similarly to ARL3-Y90C, behaving as a fast-cycling GTPase, and therefore leading to adRP. Since intracellular GTP concentrations are 10 times higher than GDP, fast-cycling GTPases usually reside in their active conformation [46]. The ARL3-Y90C variant has strong binding to its GEF ARL13B, which impedes the binding of wild-type ARL3 to ARL13B [43]. In addition, ARL3-Y90C binds GTP more efficiently than endogenous wild-type ARL3 [43]. Our co-immunoprecipitation experiments showed that the ARL3-G70E variant does not have constitutive activity like the ARL3-Q71L variant, which makes a stable complex with RP2, suggesting that ARL3-G70E is not constitutively active. This was also observed for the ARL3-Y90C variant in GST-PDEδ pull down assays, whereas the ARL3-D67V variant showed increased binding like ARL3-Q71L [43]. Stability studies also indicate that ARL3-G70E is stable and not degraded, ruling out the stability problem reported for the ARL3-R99I variant [8]. Furthermore, we observed reduced trafficking of PDE6α to the outer segments in the ARL3-G70E hiPSCs-ROs. Therefore, for ARL3-G70E, in addition to deficient ciliary INPP5E, altered PDE6δ and UNC119 function caused by aberrant ARL3-GTP cycling within the cell could also contribute to retinal degeneration.

### The effect of ARL3-G70E in photoreceptor nuclei positioning

A secondary effect of the ARL3-Q71L and ARL3-Y90C variants observed in mice was the altered positioning of photoreceptor nuclei during retinal development, displaced from the outer nuclear layer to the inner nuclear layer [43, 44]. Overexpression of ciliary lipidated cargos INPP5E and NPHP3 was sufficient to rescue the ARL3-Y90C migration defect [43]. Dynein is important for positioning of nuclei during photoreceptor development [47]. Since ARL3 and dynein light chain LC8 induce dissociation of dynactin from the dynein motor [48], ARL3-G70E could alter the dynactin-dynein interaction and thereby nuclear positioning. We have been unable to investigate photoreceptor nuclear migration in our hiPSCs-RO model, given that some rod and cone photoreceptors do not migrate properly even in control organoids, and remain in the inner nuclear layer. Further studies of the ARL3-G70E variant in a mouse model would be required to investigate its effect on nuclear migration.

### ARL3-G70E may lead to a negative regulation of sonic hedgehog (Shh) signaling through INPP5E

INPP5E plays an important role in the cilium by controlling cilia growth and PI3K signaling [49]. Importantly, variants in *INPP5E* are associated with ciliopathies, including Joubert syndrome and syndromic and non-syndromic IRD [49–51]. Strikingly, Hanke-Gogokhia and colleagues initially did not observe INPP5E in the outer segments or apical inner segments of mouse photoreceptors, instead localizing the protein to the Golgi [14]. More recently, the same group showed the localization of INPP5E to the photoreceptor inner segments and connecting cilium, excluded from the outer segments [52]. The specificity of the connecting cilium staining was confirmed, as it was absent in *^ret^Inpp5e^−/−^* photoreceptors. In accordance with this, using the same commercial antibody, we observed INPP5E localization in the cilia of fibroblasts, hiPSCs-RPE, and photoreceptor connecting cilium of hiPSCs-ROs (Fig. 4 and Supplementary Fig. S2). We did not observe INPP5E in the Golgi of the control or ARL3-G70E retinal organoid photoreceptors (Supplementary Fig. S2**).**

Trafficking of INPP5E to the cilium in PDEδ patient fibroblasts has been proposed to occur through binding to the solubilizing factor PDEδ in the cytosol [22, 53]. Once in the cilium, ARL3-GTP binding to PDEδ stimulates the release of INPP5E [9, 38, 39]. However, genetic ablation of PDEδ did not appear to affect INPP5E localization in the retina [52], such that the factors that control INPP5E photoreceptor cilia traffic still remain to be fully determined. INPP5E participates in the ciliary composition of phosphoinositides, which is made of high phosphatidylinositol-4-phosphate (PtdIns (4)P) levels, and low phosphatidylinositol 4,5-bisphosphate (PtdIns(4,5)P_2_) levels. Balancing these levels has an impact on proper Sonic hedgehog (Shh) signaling [54], which is important for ocular development in general and photoreceptor differentiation in particular [55, 56].

The levels of INPP5E were reduced in the conditional *Arl3* knock-out mouse retina, but the Golgi localization of ARL3 was unaffected [14]. We observed a reduction of INPP5E in the cilia of the ARL3-G70E patient-derived hiPSCs-RPE and hiPSCs-ROs. Importantly, retina-specific knock-out of *Inpp5e* in mice leads to impaired photoreceptor axoneme extension and rapid retinal degeneration [52]. Less ciliary INPP5E would imply higher PtdIns(4,5)P_2_ and phosphatidylinositol (3,4,5)-trisphosphate (PI(3,4,5)P_3_) levels in the cilium, and a negative regulation of Shh signaling, affecting cilium homeostasis and photoreceptor differentiation. This reduction in INPP5E was not observed in fibroblasts (Fig. 4), suggesting the presence of specific ARL3 interactors in RPE and photoreceptor cells that influence INPP5E delivery. This underscores the critical role of ARL3 in retinal function, though further investigations will be needed to explore this in detail.

### General conclusion

In summary, we have identified a monoallelic novel missense variant G70E in *ARL3* that is associated with non-syndromic dominant retinal dystrophy with full penetrance and variable clinical expressivity. We modeled this genotype to study its molecular consequences and cellular effects using fibroblasts, hiPSCs-RPE, and hiPSCs-ROs. Our data highlights the importance of ARL3 for retinal homeostasis, making it the first investigation of ARL3 dysfunction in patient-derived and retinal stem cells. Further research, including studies in a mouse model, could help to fully elucidate the impact of this *ARL3* variant on retinal function.

## Materials and methods

### Clinical assessment

Ten affected patients from five unrelated families underwent full ophthalmic examination including color fundus photography and autofluorescence (AF) imaging. Family 1, individual II.2; Vision (logMAR notation), Color vision, Goldmann kinetic visual fields, full-field electroretinography (ERG), fundoscopy, blue-light autofluorescence imaging, on near-infrared-light autofluorescence imaging and optical coherence tomography (OCT) of the macula. For other patients, AF imaging (HRA2, Heidelberg Engineering, Heidelberg, Germany), OCT (Spectralis HRA2, Heidelberg Engineering, Heidelberg, Germany) and Goldmann perimetry was obtained. Full field ERG was performed according to International Society for Clinical Electrophysiology of Vision (ISCEV) standards in all patients. Clinical data are summarized in Table 1.

### Whole exome sequencing (WES) for Family 1

Genomic DNA was extracted from blood samples utilizing the MagCore Genomic DNA Whole Blood Kit. For exome sequencing, the exomes were enriched using the SureSelect Human All Exon V6 kit (Agilent) and sequenced on a NextSeq500 system (Illumina). The resulting sequence reads were aligned to the human reference genome (build hg19/GRCh37), and variant calling was conducted using the CLC Genomics Workbench (version 7.5.4). Variant annotation was performed with Alamut HT (version 1.1.5) software, and the variants were filtered against a comprehensive list IRD genes from the RetNet gene panel (version 5), which includes 290 genes (list available upon request).

Then, variants were filtered to exclude those with a minor allele frequency (MAF) of 2% or greater, based on data from [57–59], the 1000 Genome Project [60], and an in-house sequence variation database (Exomes of Ghent). The remaining truncating variants, including nonsense, frameshift, and splice site mutations, along with missense variants predicted to be pathogenic (SIFT<0.05, PolyPhen-2 > 0.8, Align GVGD>C25, Grantham>60), were retained and further analyzed using Alamut Visual. Variants identified as potentially pathogenic were validated by Sanger sequencing, employing the BigDye Terminator v3.1 kit (Life Technologies). The primers used for Sanger sequencing were: forward, 5'-CCTGCTAAATTCACAGTGATTAAT-3′ and reverse, 5'-ATCACATATGGCAGAGTACTTT-3′. Segregation analysis was also performed using Sanger sequencing with the same primers. Each reaction included both a positive and negative control.

### Whole exome sequencing (WES) for Family 3

Genomic DNA of the proband was extracted from whole blood. Clinical genome sequencing was performed on probands’ genomic DNA using TruSight One Expanded (Illumina) and paired-end sequencing on a NextSeq 500 platform (Illumina) (Clinical Genomics Lab, Bern University Hospital). Sequence alignment and local realignment to the human reference genome (GRCh37hg19) were performed by CLC Genomics Workbench v.12.0.2 (Qiagen Bioinformatics). Variants with a frequency below 5% in coding regions of 264 genes associated with retinal dystrophy (list provided on request), including flanking regions (±8 bp) were evaluated using OMIM, dbSNP153, gnomAD v2.1.1, ClinVar, HGMD Professional, LOVD and Varsome. The reported *ARL3* variant (NM_004311.4) was confirmed by Sanger sequencing in the proband and analyzed in available family members (PCR primers and amplification program available on request).

### Whole exome sequencing (WES) for Families 2, 4, and 5

Genomic DNA was extracted from whole blood or saliva samples following standard procedures. WES was performed on probands’ genomic DNA at CeGaT GmbH, (Tübingen, Germany). There, sequencing libraries were generated using the Twist Human Core Exome kit (Family 2:IV.1; Twist Bioscience) or Twist Human Core Exome Plus kit (Family 4:II.2 and Family 5:I.1; Twist Bioscience), following manufacturer’s protocols. Libraries underwent paired-end sequencing on a NovaSeq 6000 (Novogene, CeGaT) platform (Illumina), resulting in sequences of 100 bases. Raw sequence files were assessed, trimmed, and finally mapped back to the human genome reference sequence (build hg19/GRCh37), using BWA (v0.7.17). Then, Picard (v2.14.0-SNAPSHOT) and GATK (v4.1.4.1) [61], were used to process mapped reads and perform base quality score recalibration and variant calling. DNA variants were processed and scored according to an internal computational pipeline [57] using ANNOVAR [62] with the addition of MutScore [63]. The resulting variants were then classified based on their molecular profile (nonsense, frameshift, missense, and splice sites) and prioritized according to compatible patterns of inheritance (i.e. a homozygous, compound heterozygous state for recessive, heterozygous for dominant, or on the X chromosome, for X-linked inheritance) and to their presence within the list of genes currently associated with IRDs in the RetNet database (https://retnet.org).

### Segregation analysis for Families 2 and 5

Segregation confirmation of the NM_004311.3: c.209G > A, p.(Gly70Glu) variant was performed for the available family members via polymerase chain reaction (PCR) using the GoTaq polymerase (Promega), 2 ng of template DNA and the following primer pair: 5’-ACAACCTGGATGAAACAAGCA-3′ (CR-7213) and 5’-TCTTCTGACTTCCCTTCTGCT-3′ (CR-7214). PCR products were purified using ExoSAP-IT (ThermoFisher) and Sanger sequencing was performed by Microsynth (Balgach, Switzerland). Sequences were visualized and compared to the *ARL3* gene’s reference sequence (Ensembl, GRCh37) [64] with the CLC Genomics Workbench 12 software (QIAGEN).

### Homozygosity mapping

Regions of homozygosity (ROH) were detected from the WES data by using AutoMap [65].

#### AlphaFold structure prediction

Initial structural models for wild-type ARL3 were generated using AlphaFold3, with the input sequence obtained from the UniProt entry for ARL3 (ID: P36405). For the mutant (G70E) model, the point mutation was manually introduced into the input sequence prior to AlphaFold3 prediction. GDP, GTP, and Mg^2+^ were incorporated into the structures as part of the AlphaFold inputs.

The predicted structures were refined through energy minimisation using GROMACS with the CHARMM36 force field. During this process, solvent molecules were added, and steric clashes were resolved. Distance-based interactions were validated using PyMOL's measurement tools and cross-checked with Mol* to ensure plausible binding interactions and consistency across visualisation platforms. Final structural figures were styled and rendered in PyMOL for clarity and presentation quality.

### Fibroblasts

A skin biopsy was collected from individual II.2 (Family 1) using a biopsy punch (Stiefel) and was transferred to a 15 ml conical tube containing fibroblast medium. ARL3-G70E patient fibroblasts and control human dermal fibroblasts of neonatal origin (HDFn) were maintained in fibroblast media (Dulbecco's Modified Eagle's Medium (DMEM)/F12, 10% Fetal Bovine Serum (FBS) + 1% Penicillin–Streptomycin (Pen/Strep) + 1% Sodium pyruvate). To induce cilia growth, fibroblasts were incubated with serum starvation medium (DMEM/F12, 0.2% FBS + 1% Pen/Strep +1% Sodium pyruvate).

### hiPSCs generation from fibroblasts

*ARL3* c.209G > A patient fibroblasts were Nucleofected using Cell Line Nucleofector Kit R (Lonza) containing 1 μg of the episomal reprogramming vectors pCXLE-hOCT3/4-shp53-F (Addgene plasmid 27 077), pCXLE-hUL Addgene #27080), pCXLE-hSK (Addgene #27078) and pSimple-miR302/367 (Addgene #98748), as previously described [66].

### hiPSCs differentiation to RPE and retinal organoids

hiPSCs were differentiated to RPE based on a method previously described [67]. Briefly, hiPSCs were seeded on Geltrex-coated (Thermo Fisher) six-well plates with mTESR Plus Medium (Stem Cell Technologies) until reaching full confluency. On day 0, cells were cultured with neural induction media (1:1 DMEM/F12/GlutaMAX™ (Gibco™), neurobasal medium (Gibco™), 55 μM 2-mercaptoethanol, 0.5 × B27, 0.5 × N2, 1x L-Glutamine and 1 × Anti-Anti). On day 7, cells were cultured in RPE medium (DMEM, GlutaMAX™, 10% knock-out serum replacement (Gibco™), 1x non-essential amino acids, 1 × L-Glutamine, 55 μM 2-mercaptoethanol (Gibco™), 1 × Anti-Anti. Fresh 50 ng/ml recombinant human activin A (PeproTech) was added with every media change. When the first signs of pigmentation became visible to the naked eye, activin A supplementation was stopped. hiPSCs were differentiated into retinal organoids as previously described [68, 69].

### Immunofluorescence

RPE cells were fixed in 2% paraformaldehyde (PFA) for 15 min at room temperature (RT), followed by 1% Triton-X-100 treatment for 5 min and blocking in 2% fetal bovine serum (FBS) for 20 min. Subsequently, cells were incubated with primary antibodies diluted in a blocking solution for 1 h. After incubation, cells were washed three times in phosphate buffered saline (PBS) and incubated with their corresponding Alexa Fluor conjugated secondary antibodies (ThermoFisher). After secondary antibody incubation, DAPI (ThermoFisher) was incubated for 5 min. Finally, slides were washed three times in PBS for 5 min and mounted in DAKO fluorescence mounting medium (Agilent, Santa Clara, CA, USA).

### Cryopreservation and Immunohistochemistry of retinal organoids

Retinal organoids were briefly washed once in PBS and placed in a mixture of 4% PFA + 5% sucrose in PBS for 40 min. Organoids were then placed in 6.25% sucrose in PBS for 1 h, followed by 12.5% sucrose in PBS for 30 min and in 25% sucrose in PBS for 1 h. All incubation steps were performed at 4°C. Organoids were then embedded in OCT, slowly frozen on dry ice, and stored at −80°C until cryosectioning. Cryosections (7 μm thick) were collected and stored at −20°C for later analysis.

### Immunohistochemistry

Sections were first washed once in PBS and blocked with 10% donkey serum, 0.05% Triton X-100 in PBS for 30 min. The primary antibodies used are listed on Supplementary Table S2. The primary antibodies were incubated in the blocking solution diluted 50% in PBS for 1 h. Sections were then washed in PBS and incubated with Alexa Fluor (ThermoFisher) secondary antibodies in the diluted blocking solution for 45 min. Nuclei were visualized using DAPI (2 μg/ml). Samples were washed in PBS and mounted using DAKO fluorescence mounting medium (Agilent).

### Cilia quantification and statistical analysis

Images were obtained using a Leica Stellaris 8 confocal microscope. At least 3 images were taken for each condition. For hiPSC-RPE cells and fibroblasts, cilia were segmented and cilium length was quantified using the plugin CiliaQ on Fiji/ImageJ based on the ARL13B channel (and basal body present in the PCN channel) [70]. Manual counting was performed to measure photoreceptor ciliation and cilia length in retinal organoids. For colocalization analyses in retinal organoids, the outer segment region was selected and the Manders’ colocalization coefficient was obtained using JACoP on ImageJ. Statistical analyses were carried out using Prism and are indicated at the bottom of each figure’s legend.

### Cell culture and transfection

Human HEK293 cells were cultured in DMEM supplemented with 10% FBS and 1% Pen-Strep in a 5% CO2 humidified incubator at 37°C. 2 × 10^5^ cells were seeded in 6-well plates and 0.5 μg of GFP-RP2 plasmid and 2 μg of VSV-ARL3-WT, VSV-ARL3-Q71L, and VSV-ARL3-G70E plasmids were co-transfected using Lipofectamine 2000 (Invitrogen) in a non-antibiotic medium. 5 h post-transfection, the medium was replaced with a fresh complete medium, and the cells were incubated at 37°C for 48 h.

#### Plasmids

VSV-ARL3-WT and VSV-ARL3-Q71L were generated by site-directed mutagenesis in a pcDNA4/TO/VSV plasmid that adds a VSV-G epitope tag to the C-terminus of ARL3, as previously described [32]. VSV-ARL3-G70E was generated by site-directed mutagenesis to introduce the G70E change in ARL3.

### Co-Immunoprecipitation assay

Transfected HEK293 cells were resuspended in iced-cold lysis buffer (10 mM Tris/Cl pH 7.5, 150 mM NaCl, 0.5 mM EDTA, 0.5% Nonidet™ P40 Substitute) and 5% protease inhibitor cocktail (Merck Life Science UK Limited) and incubated 30 min on ice. Protein extracts were recovered after centrifugation. After saving 10% of the supernatant as input, lysates were incubated with pre-equilibrated GFP-Trap Magnetic Agarose beads (Chromotek, gtma-20) following the manufacturer’s instructions. Bound proteins were eluted from the beads by boiling for 5 min with protein SDS-loading buffer.

### Western blot

Proteins were analyzed by 12% SDS-PAGE and transferred onto PVDF membranes. Protein membranes were blocked with 5% non-fat milk in PBS containing 0.1% Tween 20 for 1 h and incubated overnight at 4°C with primary antibodies: mouse anti-GFP (Proteintech, 66 002–1-Ig) and mouse anti-VSV (Sigma Aldrich, SAB4200695). After incubation with HRP-conjugated secondary antibodies for 1 h at room temperature, proteins were detected using Clarity Western ECL substrate (BioRad) and imaged with ImageLab on a BioRad ChemiDoc XRS+. The ImageJ software was used for quantification.

### Statistical analysis

Statistical significance of data, equal standard deviation (SD) and normal distribution were first assessed using Bartlett and Shapiro–Wilk tests. As data followed a normal distribution and showed homogeneity of variance, parametric T-test was used for statistical significance analysis. Analysis was performed using GraphPad Prism 9.

## Supplementary Material

HMG_2024_OA_00888_R1_Supplementary_Material_ddaf029
