# Supplementary material for: A novel recurrent ARL3 variant c.209G > A p.(Gly70Glu) causes variable non-syndromic dominant retinal dystrophy with defective lipidated protein transport in human retinal stem cell models
Source: Hum Mol Genet. 2025 Mar 3;34(9):821–34. doi: 10.1093/hmg/ddaf029 (PMC12010153; doi:10.1093/hmg/ddaf029)
Supplement: HMG_2024_OA_00888_R1_Supplementary_Material_ddaf029 [file hmg_2024_oa_00888_r1_supplementary_material_ddaf029.docx]

**A novel recurrent ARL3 variant c.209G>A p.(Gly70Glu) causes variable non-syndromic dominant retinal dystrophy with defective lipidated protein transport in human retinal stem cell models**

**Supplementary material**

**
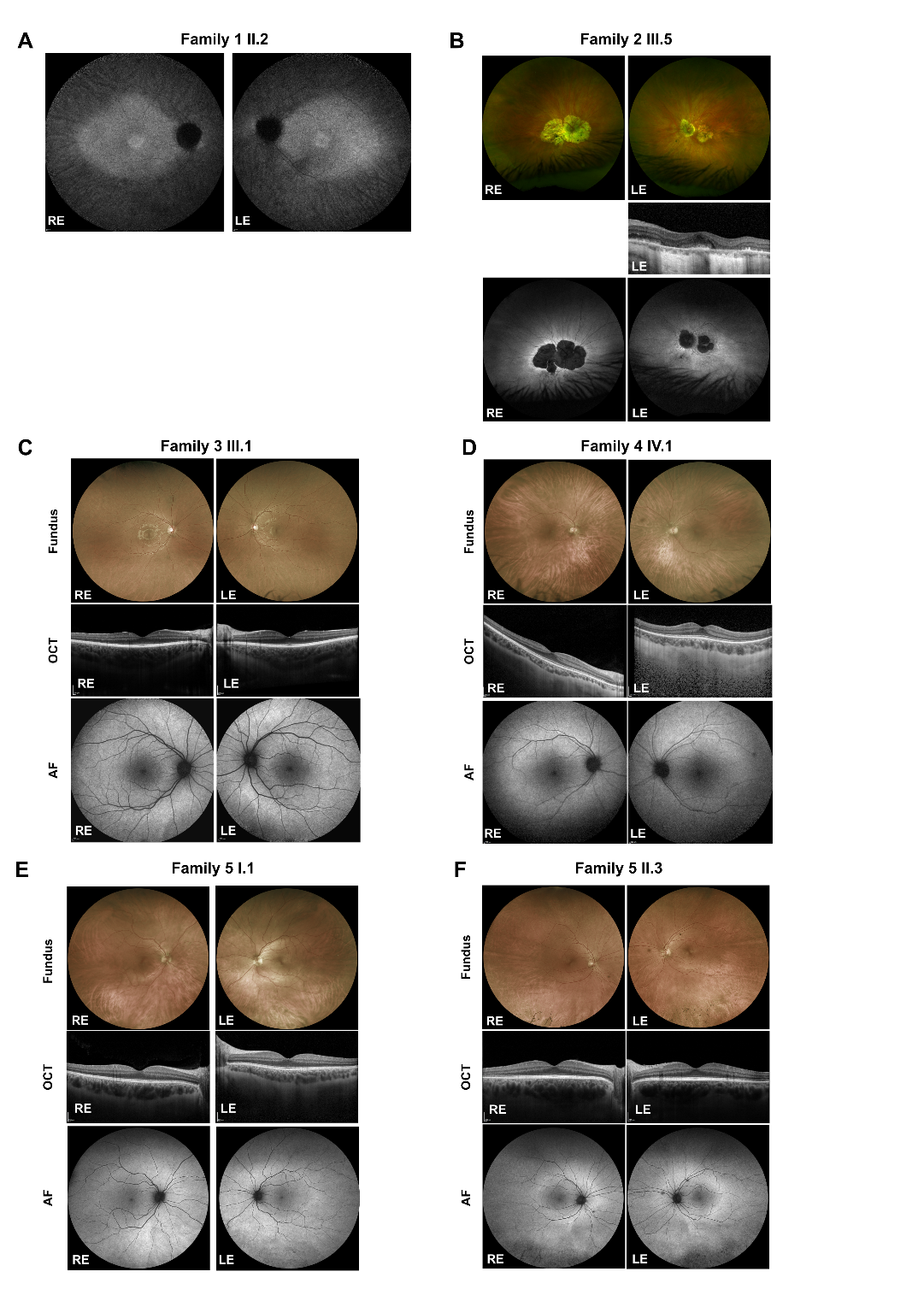
**

**Supplementary Figure S1. A.** Near-infrared-light autofluorescence of Family 1 individual II.2, showing a macula-centered rhomboid area of preserved autofluorescence signal with a parafoveal ring of mildly reduced signal. Outside of this rhomboid, the autofluorescence signal is severely reduced. **B**. Family 2, individual III.5 shows large areas of hypoautofluorescence on AF and fundus color. OCT of the left eye confirms the thinning of the outer retinal perifoveal layers. **C**. Family 3, the son (individual III.1) has a hyperautofluroescent ring that can be seen developing, with OCT showing thinner ONL outside fovea, and changes on fundus imaging. **D**. Family 4, the granddaughter (individual IV.1) has a normal appearance of fundus with reduced ONL outside the fovea on OCT and a band of hyperautofluorescence beyond the arcades. **E**. Family 5, the mother (individual I.1) is mildly affected, with a hyperautofluorescent ring on AF imaging. **F**. Family 5, the 34-year-old sister (individual II.3) has a hyperautofluorescent ring around the fovea, with some hypoautofluorescent areas beyond arcades. The fundus color shows some bone spicules at the peripheral retina. Abbreviations used: AF: autofluorescence; OCT: optical coherence tomography; ONL: outer nuclear layer.


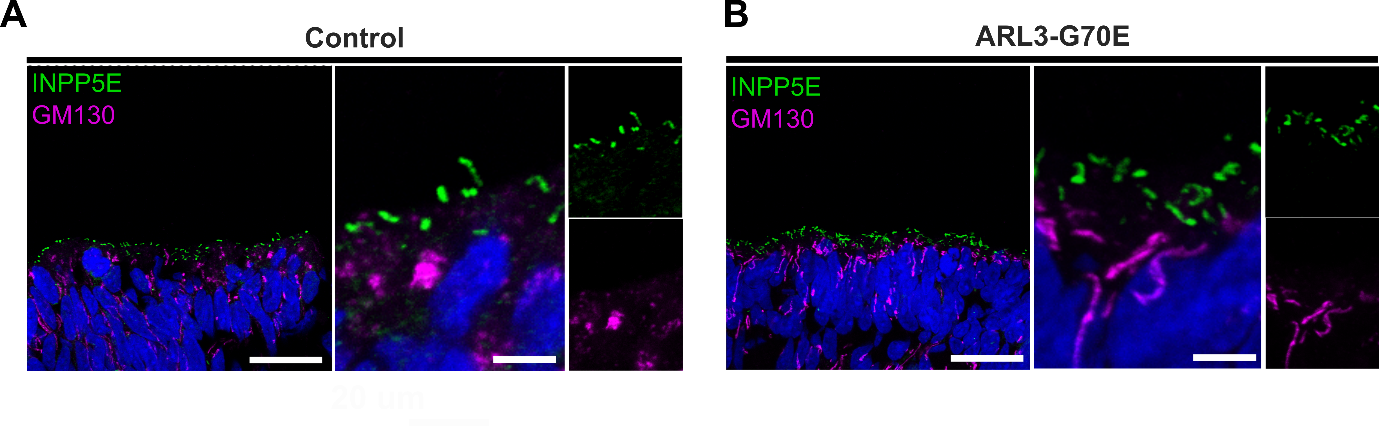


**Supplementary Figure S2. ARL3-G70E hiPSCs-ROs do not show major differences in INPP5E or Golgi localization.** Immunofluorescence of control ROs (**A**) and ARL3-G70E ROs (**B**) at day 120 of differentiation, stained with INPP5E (green) and the Golgi marker GM130 (magenta). Nuclei are marked with DAPI (blue). No major differences were observed between controls and ARL3-G70E in the localization of INPP5E or GM130. Scale bars: 20 µm (left panel) and 5 µm (inset, right panel).

**
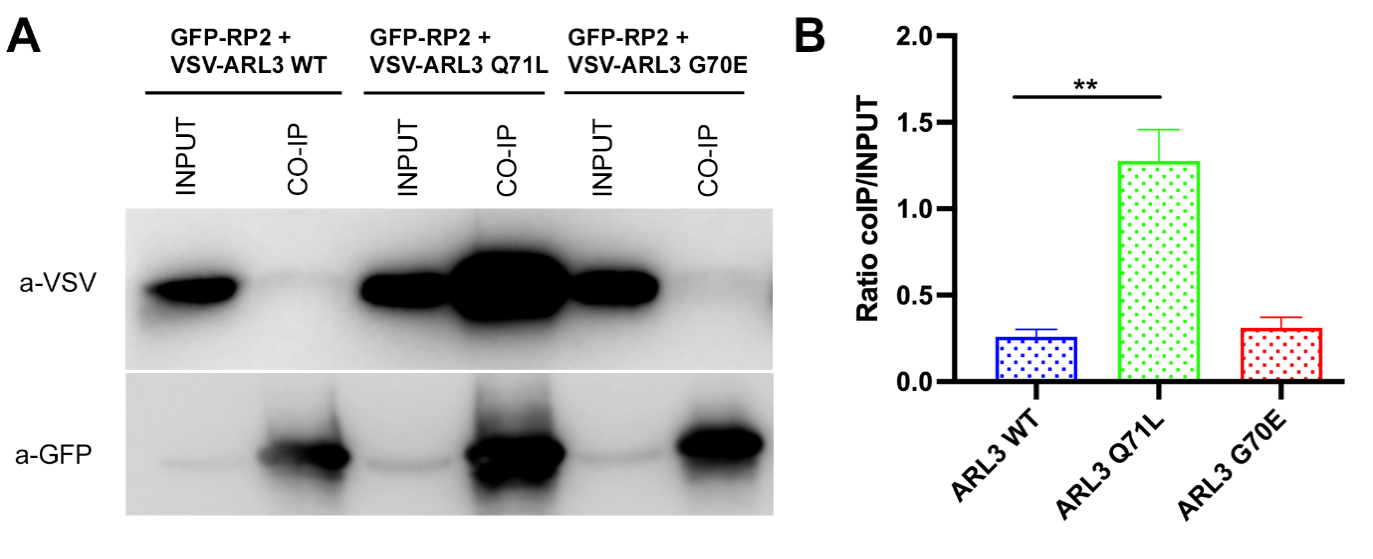
**

**Supplementary Figure S3. Co-immunoprecipitation experiment of ARL3 with its GAP RP2.** GFP immunoprecipitation from HEK293T lysates expressing GFP-RP2 and ARL3-VSV variants. **A**. For each combination of transfected plasmids, western blots show 10% of lysate input and 30% of the GFP-trap eluates. **B**. The ratio of co-immunoprecipitated protein to input is shown for each ARL3 variant (n≥5), ** p<0.01.

|  | **Supporting (PP1-PP5)** | **Moderate (PM1-PM6)** | **Strong (PS1-PS4)** | **Very Strong (PVS1)** |
| --- | --- | --- | --- | --- |
| **Population data** |  | Absent in population databases (PM2) |  |  |
| **Computational and predictive data** | Multiple lines of computational evidence support a deleterious effect on the gene (PP3) |  |  |  |
| **Functional data** |  |  | Well-established functional studies show a deleterious effect (PS3) |  |
| **Segregation data** |  | Segregation with disease in multiple affected family members (PM1) |  |  |
| ***De novo* data** |  |  | *De novo* (paternity and maternity confirmed) (PS2) |  |
| **Other data** | Patient’s phenotype highly specific for gene (PP4) |  |  |  |
| **Summary** | **2** | **2** | **2** | **0** |

**Pathogenicity**

**Classification: Pathogenic (2 Strong), 2 Moderate, 2 Supporting; Total 14 points**

**Supplementary Table S1.** ACMG variant classification for the *ARL3* variant c.209G>A, p.(Gly70Glu).

| Antibody | Species | Supplier | Catalog number | Dilution |
| --- | --- | --- | --- | --- |
| PDE6A | Rabbit | Proteintech | 21200-1-AP | 1:1000 |
| Rhodopsin | Mouse | Millipore | MABN15 | 1:1000 |
| Acetylated tubulin | Mouse | Sigma-Aldrich | T6793 | 1:1000 |
| L/M-Opsin | Rabbit | Millipore | 5405 | 1:500 |
| INPP5E | Rabbit | Proteintech | 17797-1-AP | 1:100 |
| GM130 | Mouse | BD Transduction LB | 610823 | 1:1000 |
| ZO-1 | Rabbit | Thermo Fisher | 61-7300 | 1:200 |
| PMEL17 (Melanosome Clone HMB45) | Mouse | Agilent | M0634 | 1:100 |
| ARL13B | Rabbit | Proteintech | 17711-1-AP | 1:1000 |
| PCN | Mouse | Abcam | ab28144 | 1:1000 |

**Supplementary Table S2.** List of primary antibodies used in immunofluorescence (IF).
